# An updated meta-analysis of the relationship between vitamin D levels and precocious puberty

**DOI:** 10.3389/fendo.2023.1298374

**Published:** 2023-12-05

**Authors:** Hong Cheng, Dan Chen, Hui Gao

**Affiliations:** ^1^ School of Medical Nursing, Fuyang Vocational and Technical College, Fuyang, Anhui, China; ^2^ Maternal and Child Health Care, School of Public Health, Anhui Medical University, Hefei, Anhui, China; ^3^ Department of Pediatrics, the First Affiliation Hospital of Anhui Medical University, Hefei, Anhui, China

**Keywords:** precocious puberty, vitamin D, meta-analysis, systematic review, idiopathic central precocious puberty in girls

## Abstract

**Background:**

Some studies have investigated the association between vitamin D levels and precocious puberty (PP) but with limited sample sizes and inconsistent conclusions across studies.

**Methods:**

Until July 2022, a comprehensive electronic search of works of literature was conducted in MEDLINE, Web of Science, and CNKI (Chinese National Knowledge Infrastructure). A systematic review and meta-analysis of 15 case-control studies with 2145 cases and 2063 controls was conducted to explore the relationship between vitamin D and PP. Stratified analyses by year of publication, country, diagnosis category of PP, child’s sex, and methods of 25(OH)D test were conducted.

**Results:**

There was a negative correlation between 25(OH)D concentrations and PP in all study populations (SMD = -1.046, 95%CI = -1.366, -0.726). The pooled SMD remained significant in Chinese studies (SMD = -1.113, 95%CI = -0.486, -0.741), studies published before or after 2018 (SMD = -0.9832 and -1.185, 95%CI = -2.044, -1.133 and -1.755, -0.726), studies with female children (SMD = -1.114, 95%CI = -1.446, -0.781), and studies using electrochemiluminescence to detect 25(OH)D (SMD = -0.999, 95%CI = -1.467, -0.531). Vitamin D deficiency also increased the risk of PP (OR = 1.531, 95%CI = 1.098, 2.134). Unfortunately, heterogeneity was high in all analyses, and there was some publication bias.

**Conclusion:**

This systematic review and meta-analysis demonstrated an association between vitamin D and precocious puberty. We recommend more high-quality studies, especially prospective cohort studies with big sample sizes or some randomized controlled intervention trials, to validate the reliability of the results.

## 1 Introduction

Precocious puberty (PP) is usually defined as puberty, which includes the acquisition of secondary sexual characteristics, that starts before 8 years of age for girls or before 9 years of age for boys (1). Most of PP in girls is idiopathic central PP (ICPP). Previous studies have found that 10% of white girls and 23% of Black girls in the United States have started their puberty before age 8 (2). Compared with data from the 1960s, the current breast development occurs earlier (3, 4). The reasons for PP care are mainly divided into two groups. One is as a result of the “early commencement of pulsatile secretion of gonadotrophin-releasing hormone (GnRH)” and another is primarily “related to increased sex steroid production, independent of GnRH” (5). Earlier puberty in girls may be one of the important risk factors for an increasing prevalence of childhood obesity (6), which is another substantial public health question. In addition, earlier puberty may accelerate the premature fusion of epiphyses, decrease height growth potential, and increase negative psychosocial impacts including fear and inferiority (7).

Humans can get vitamin D through sun exposure or food. Vitamin D is metabolized in the liver to 25-hydroxyvitamin D [25(OH)D] and then converted to the biologically active form of 1,25-dihydroxyvitamin D [1,25(OH)_2_D] in the kidneys. Concentrations of 25(OH)D, the more robust form compared with 1,25(OH)_2_D, in serum reflect human vitamin D status. Vitamin D plays a key role in bone and mineral metabolism. Evidence has shown that 25(OH)D levels are associated with a variety of diseases, including immune dysfunction, obesity (8, 9), metabolic syndrome, insulin resistance (10), infection (11), cancer, and cardiovascular abnormalities (12, 13). Several studies have also shown a correlation between vitamin D status and time to menarche (14, 15) and central precocious puberty (CPP) (16–18). Previously, only a short communication performed a meta-analysis focused on the association between vitamin D deficiency (usually <20 ng/mL) and PP by including six studies (19). Systematic reviews and meta-analyses have been recognized as the best means of objectively evaluating and synthesizing research evidence on a particular issue and are generally considered the highest level of evidence. Considering that there are a few relevant studies with a small sample size, we further performed a comprehensive search to include more studies to conduct a systematic review and, if necessary, a meta-analysis, to further provide evidence for the association between vitamin D and PP.

## 2 Materials and methods

**Figure 1:**
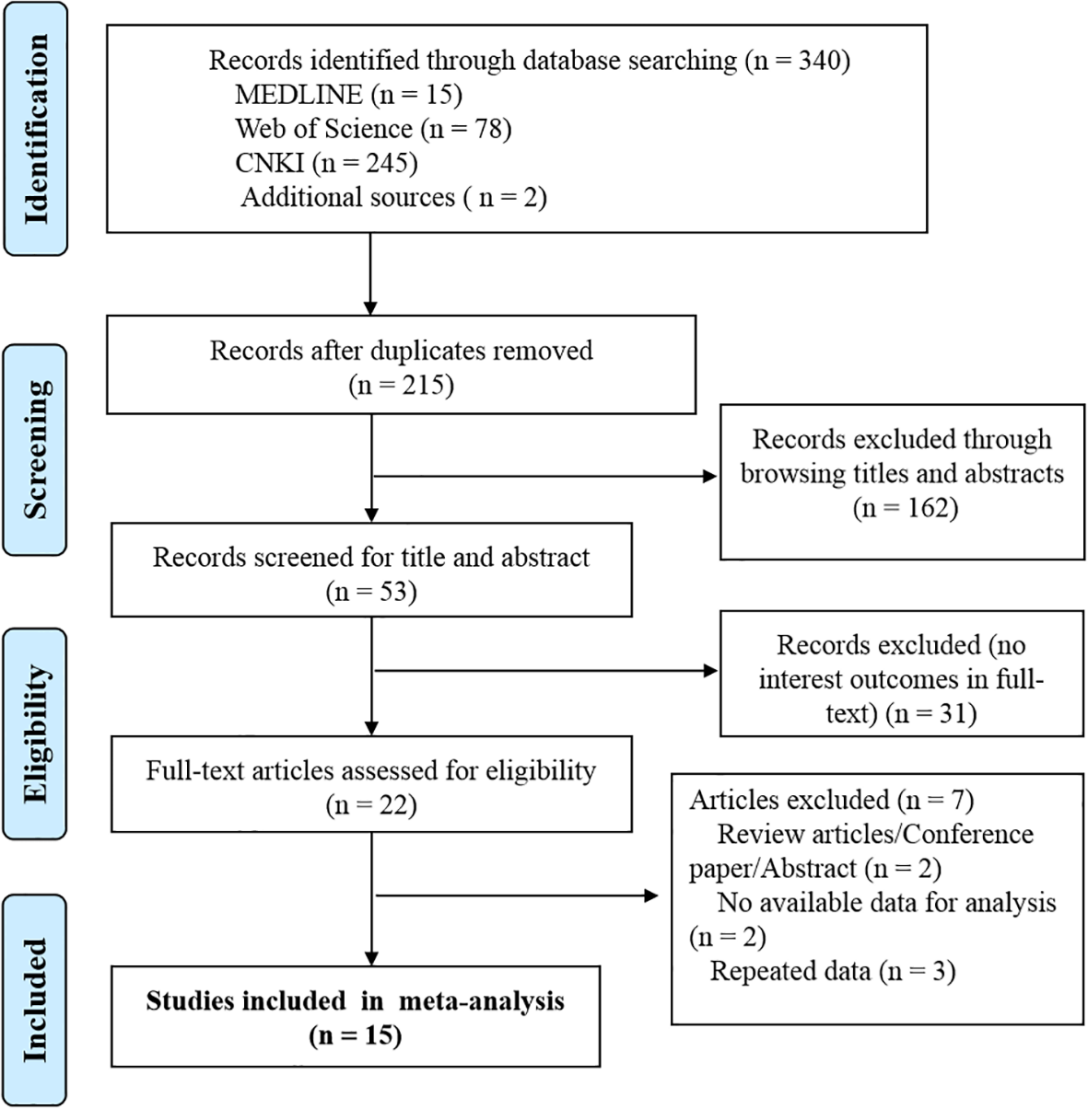
Flow diagram for the process of selecting the final 15 studies.

This meta-analysis is reported in accordance with the Preferred Reporting Items for Systematic Reviews and Meta-Analyses (PRISMA) guidelines. We used a PRISMA flow chart to visualize the selection, sorting, and review process (**Figure 1**).

### 2.1 Literature search

We searched the MEDLINE (through PubMed), Web of Science, and China National Knowledge Infrastructure (CNKI) electronic databases. We conducted a comprehensive literature search for studies published up until 2022, without any restriction on the earliest publication date. Search terms used were as follows: (“vitamin D” OR ergocalciferol OR cholecalciferol OR “25 hydroxyvitamin D” OR 25(OH)D OR “calciferol” OR “calcitriol” OR “cholecalciferol” OR “ergocalciferol” OR “hydroxycholecalciferols” OR “hydroxyergocalciferols”) AND (“precocious puberty” OR “early puberty” OR “advanced puberty” OR “premature puberty” OR “sexual precocity” OR “gonadarche” OR “thelarche” OR “pubarche” OR “adrenarche”) and relevant Chinese technical terms for the Chinese Database. We only considered human studies. The references cited in identified publications were also searched to locate additional studies.

### 2.2 Inclusion and exclusion criteria

The process of inclusion, following the PECOS (Population, Exposure, Comparison, Outcome, and Study design) approach, was meticulously designed to ensure a comprehensive and unbiased review. The criteria were as follows:

1. Population: Our focus was on children and adolescents within the age spectrum of 6 to 12 years. This age group was chosen due to its critical role in growth and development and the potential impact of vitamin D_3_ on these processes.
2. Exposure: We specifically looked at studies that measured the concentration of 25(OH)D_3_, a reliable indicator of vitamin D status in the body. This was considered the primary exposure of interest given its direct relevance to our research question.
3. Comparison: To provide a meaningful context for our results, we compared the data from our primary population with those of healthy controls. This allowed us to discern patterns and potential deviations linked to 25(OH)D_3_ concentration.
4. Outcome: The primary outcome we focused on was PP. This outcome was selected due to its significant implications for the health and well-being of the population under study.
5. Study design: In an effort to capture a broad range of data, we included all observational studies in our review. This encompassed cohort studies, case-control studies, and cross-sectional surveys, each of which offers unique insights into the relationship between 25(OH)D_3_ concentration and PP. We excluded studies that were reviews, meetings, systematic reviews, meta-analyses, and comments. Repeated data were also excluded except for the latest one.

This rigorous approach to study selection ensured a comprehensive, relevant, and high-quality pool of data for our meta-analysis.

### 2.3 Data abstraction

According to a prespecified standard spreadsheet, two authors (Cheng H and Chen D) screened article titles and abstracts to identify studies that may be suitable for analysis. Full articles were independently assessed (Cheng H and Chen D) for final inclusion. Any discrepancy was discussed and resolved by the third author (Gao H). The information mainly consisted of the first author, year of publication, country, study design, method of vitamin D measurement, and the numbers and gender of cases and controls.

### 2.4 Statistical analysis

All analyses were performed with Review Manager 5.3. We conducted a meta-analysis of fixed effects for the association of 25(OH)D concentrations (continuous variable) and vitamin D deficiency (binary category variable) with risks of PP, obtaining mean differences (MD) and odds ratios (OR) with 95% confidence intervals (95% CIs). The I^2^ statistic and chi-square tests were used to measure heterogeneity. Heterogeneity was further explored via subgroup analyses. The symmetry of the funnel plot was visually used to evaluate the publication bias.

## 3 Results

The initial search strategy yielded 186 studies, in which 162 records were retained after removing 24 duplications. Another 121 records were also dropped, as they did not observe the association between vitamin D and CPP, after reviewing titles and abstracts. For the remaining 41 articles, 19 studies were excluded after reviewing the full text. Articles of reviews (2), no available data for outcomes of interest (2), and repeated data (3) were further excluded. Finally, 15 studies were included in the final analysis (**Figure 1**).

All 15 eligible literature were published between 2011 and 2020. Three studies were conducted in Turkey, South Korea, and Brazil, while the remaining 12 studies were conducted in China. These countries span a range of latitudes relative to the equator. China and South Korea are located in the Northern Hemisphere, with latitudes ranging from approximately 20°N to 45°N and 33°N to 43°N, respectively. Brazil, on the other hand, is located mostly in the Southern Hemisphere, spanning from approximately 5°N to 34°S, while Turkey is positioned at the junction of Europe and Asia, with latitudes ranging from approximately 36°N to 42°N. All of the studies were case-control studies. Three studies recruited case participants diagnosed with PP, four studies recruited case participants diagnosed with ICPP, and the remaining eight studies recruited case participants diagnosed with CPP. There were a total of 2145 cases and 2063 healthy children included in this meta-analysis. From all 15 eligible studies, 1665 cases and 1537 controls were girls. One study from Turkey included three cases and three controls who were boys (17). A study conducted by Meng et al. in China included 477 cases and 523 controls who were boys (20). All the remaining studies included only girls. Most studies provided mean ages among cases and controls. Except for one study that only reported the age range of the children (20), the other 14 studies indicated that the average age range for children in the case group was 6.58-11.27 years, while the average age range for children in the control group was 6.61-12.92 years. Different methods were used to detect the concentrations of 25(OH)D. Some studies also reported the number of vitamin D deficiencies among cases and controls. **Table 1** summarizes the detailed characteristics of the included studies.

**Table 1:** Characteristics of 18 case-control studies included in the present meta-analysis.

First author	Year	Country	Diagnosis of case	Sample size		Mean age		25(OH)D_3_ concentration		25(OH)D_3_ detection	Number of deficiency	
				PP	Control	PP	Control	PP	Control		PP	Control
Kaya (17)	2015	Turkey	CPP	23^a^	17^a^	8.18 ± 1.48	9.35 ± 1.65	15.17 ± 7	22.2 ± 6.1	—	18	5
Lee HS (16)	2014	Korea	CPP	60	30	8.3 ± 0.53	7.6 ± 1.3	17.15 ± 4.55	21.23± 5.07	RIA	42	13
Santos BR (21)	2011	Brazil	PP	36	97	11.27 ± 3.79	12.92 ± 2.09	18.08 ± 8.32	21.27 ± 7.03	RIA	—	—
Zhao Y (18)	2017	China	CPP	280	188	8.50 ± 0.87	8.43 ± 0.82	19.36 ± 6.15	20.98 ± 7.60	ECL	261	164
Chen H (22)	2018	China	CPP	49	49	6.55 ± 0.76	6.60 ± 0.79	20.29 ± 5.64	29.68 ± 6.17	ELISA	27	—
Huang X (23)	2019	China	ICPP	84	80	7.32 ± 1.12	7.29 ± 1.10	53.02 ± 20.64	74.32 ± 14.30	—	64	—
Liang Z (24)	2020	China	ICPP	180	160	8.40 ± 0.86	8.50 ± 0.85	59.05 ± 11.34	77.87 ± 25.03	ECL	83	30
Lu R (25)	2017	China	CPP	68	51	7.92 ± 0.21	8.04 ± 0.18	26.53 ± 3.6	35.48 ± 6.7	ECL	42	—
Meng Q (20)	2019	China	PP	998^b^	1061^c^	5~12	5~12	71.29 ± 18.867	75.79 ± 20.145	ECL	—	—
Sun J (26)	2020	China	ICPP	92	85	6.85 ± 1.05	7.02 ± 1.03	48.64 ± 8.90	61.30 ± 9.06	ICG	49	—
Tang J (27)	2015	China	CPP	82	82	—	—	21.62 ± 5.4	28.43 ± 7.1	ECL	42	—
Wang L (28)	2019	China	ICPP	31	31	8.1 ± 1.3	7.9 ± 1.2	23.03 ± 8.82	24.61 ± 7.26	ECL	—	—
Xue Z (29)	2017	China	CPP	62	62	6.58± 1.31	6.61± 1.13	20.64 ± 6.07	28.94 ± 6.91	ELISA	29	—
Yang R (30)	2017	China	PP	60	30	—	—	17.1± 4.5	21.2± 5.0	—	42	13
Zhong L (31)	2019	China	CPP	40	40	7.90 ± 0.67	7.86 ± 1.25	45.51(35.06,54.24)	60.25(52.19,72.95)	ECL	26	7

a including 3 boys.

b including 477 boys.

c including 523 boys; other studies only included girls; PP, precocious puberty; CPP, central precocious puberty; ICPP, idiopathic central precocious puberty; ECL, electrochemiluminescence; ELISA, enzyme linked immunosorbent assay; RIA, radio immunoassay; ICG, immunochromatography.

**Figure 2:**
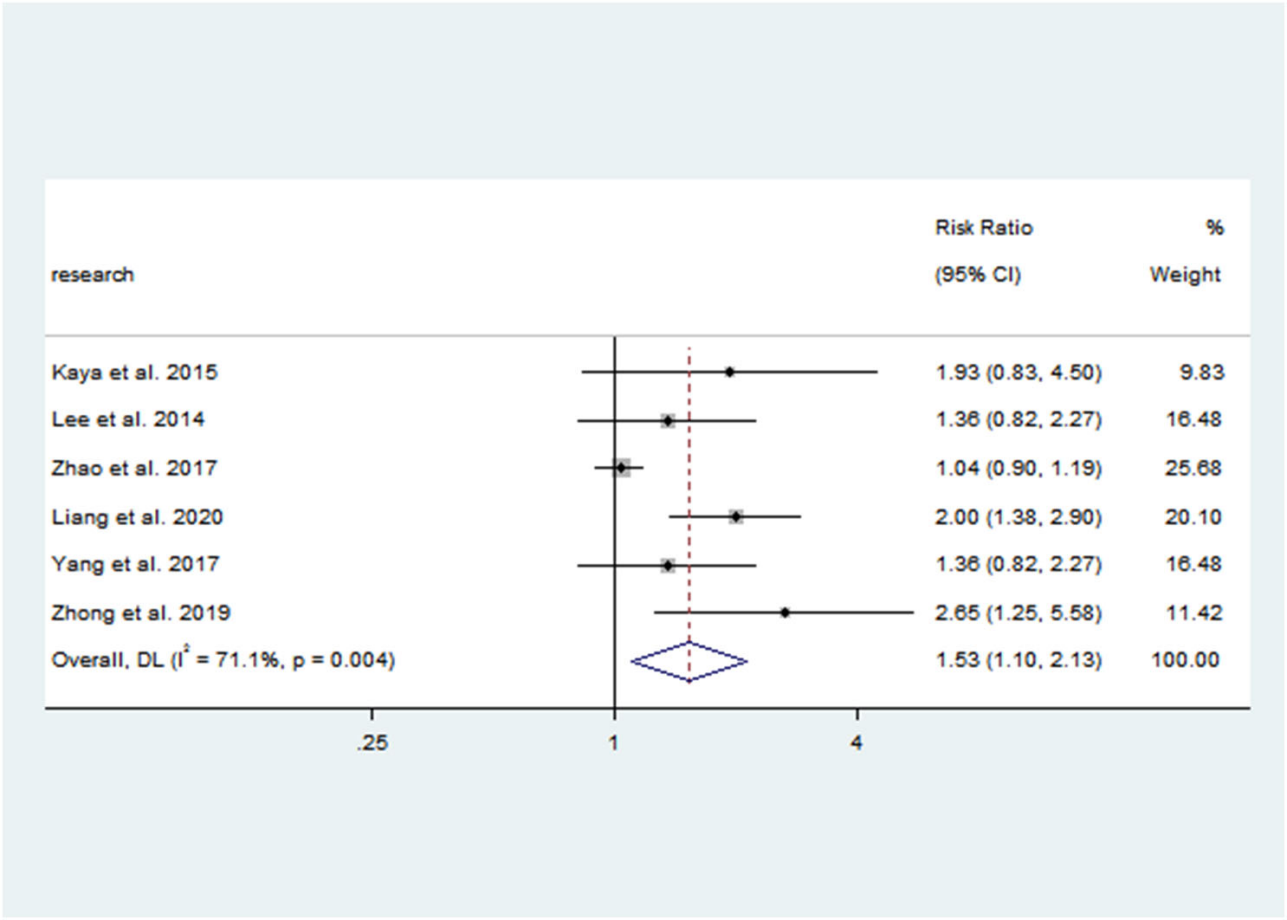
Forest plots for the association between vitamin D deficiency and PP. Results of individual and summary odds ratio (OR) estimates; 95% confidence interval (CI) and weights of each study are shown. Horizontal lines represent 95% CI, and dotted vertical lines represent the value of the summary OR.

**Figure 3:**
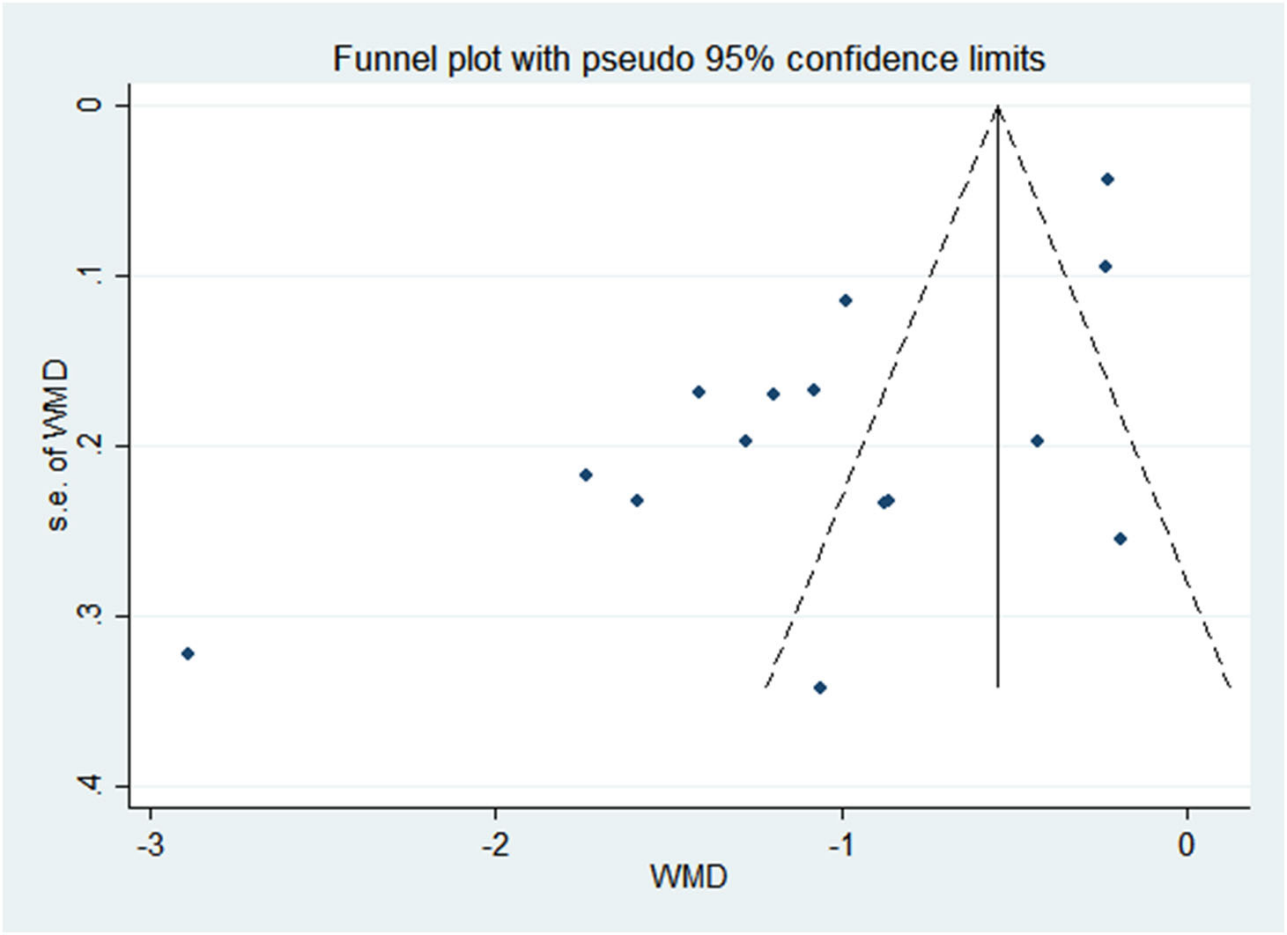
Begg’s funnel plots for the association between vitamin D and PP in overall populations.

There was a negative correlation between 25(OH)D concentrations and PP in all study populations (SMD = -1.046, 95%CI = -1.366, -0.726). Stratified by year of publication, country, diagnosis category of PP, child’s sex, and methods of 25(OH)D test, all results still suggested that the higher the 25(OH) D level, the lower the risk of PP. The pooled SMD remained significant in Chinese studies (SMD = -1.113, 95%CI = -0.486, -0.741), studies published before or after 2018 (SMD = -0.9832 and -1.185, 95%CI = -2.044, -1.133 and -1.755, -0.726), studies with female children (SMD = -1.114, 95%CI = -1.446, -0.781), and studies using electrochemiluminescence to detect 25(OH)D (SMD = -0.999, 95%CI = -1.467, -0.531). Detailed results are shown in **Table 2**. Vitamin D deficiency also increased the risk of PP by 53.1% compared with healthy children (OR = 1.531, 95%CI = 1.098, 2.134) (**Figure 2**). Unfortunately, heterogeneity was high in all stratified analyses. The heterogeneity statistic I^2^% in all stratified groups ranged from 41.9% to 96.7%. These indicated that no sources of heterogeneity were identified. In addition, the funnel plot showed that there was a publication bias (**Figure 3**).

**Table 2:** Associations between 25(OH)D and PP when examined within subgroups, and corresponding Egger’s test.

Research	No. Of studies	Analysis Model	SMD	95%CIs	Cochran’s Q (P-value)	H (95%CIs)	Heterogeneity statistic		Tau^2^	Egger’s test			
							I^2^% (95%CIs)	degree		Coef.	Std. Err.	t	P-value
All studies	15	Random	-1.046	-1.366, -0.726	252.55(<0.001)	4.247(1.765, 6.768)	94.5% (67.9%-97.8%)	high	0.357	-5.480	1.194	-4.59	0.001
Publish year													
After 2018	7	Random	-1.185	-1.755, -0.726	179.65(<0.001)	5.472(1.392,9.756)	96.7%(48.4%-98.9%)	high	0.5521	-6.893	1.872	-3.68	0.014
Before 2018	8	Random	-0.932	-2.044, -1.133	64.13(<0.001)	3.027(1.248,4.833)	89.1%(35.8%-95.7%)	high	0.2789	-5.486	2.169	-2.53	0.045
Country													
China	12	Random	-1.113	-0.486, -0.741	248.11(<0.001)	4.749(1.784,7.777)	95.6%(68.6%-98.3%)	high	0.395	-6.585	1.316	-5.00	0.001
Other	3	Fixed	-0.684	-0.954, -0.414	3.44(0.179)	1.312(1.000,2.409)	41.9%(0.0%-82.8%)	high	—	-4.321	2.603	-1.66	0.345
Category of PP													
CPP	8	Random	-1.319	-1.872, -0.766	115.41(<0.001)	4.060(1.578,6.591)	93.9%(59.8%-97.7%)	high	0.581	-8.005	1.995	-4.01	0.007
PP	3	Random	-0.460	-0.820, -0.099	8.22(0.016)	2.028(1.000-4.005)	75.7%(0.0%-93.8%)	high	0.075	-2.427	1.099	-2.21	0.270
ICPP	4	Random	-0.984	-1.375, -0.726	16.86(0.001)	2.370(1.000-4.282)	82.2%(0.0%-94.5%)	high	0.127	3.097	4.973	0.62	0.597
Only female participants	13	Random	-1.114	-1.446, -0.781	141.51(<0.001)	3.434(1.831-5.044)	91.5%(70.2%-96.1%)	high	0.332	-5.933	2.367	-2.51	0.029
ECL measures 25(OH)D	7	Random	-0.999	-1.467, -0.531	158.30(<0.001)	5.136(1.301-9.165)	96.2%(40.9%-98.8%)	high	0.363	-6.373	2.172	-2.93	0.032
Vitamin D deficiency	6	Random	1.531	1.098,2.134	17.30(0.004)	1.860(1.000-3.195)	71.1%(0.0%-90.2)	high	0.107	1.388	0.524	2.65	0.057

## 4 Discussion

In summary, a negative association between vitamin D and PP was found. Vitamin D deficiency could increase the risk of PP. However, heterogeneity was high, and the year of publication, study site, category of PP, and sex of participant could not explain the heterogeneity source. In addition, there was a publication bias and most of the included studies were published in China.

Liu et al. (2020) first performed a meta-analysis to pool the effects of vitamin D levels on the risk of PP (19). Only including six studies, they also revealed the mean difference in serum vitamin D levels between PP and healthy controls; vitamin D-deficient subjects were more likely to develop PP (19). These findings are consistent with the present study. However, a few included studies with a small sample size reduced the statistical efficiency. In addition, the meta-analysis was published as a short communication, and less information was available on relevant included research. Animal studies by Kinuta K et al. (2011) have shown that vitamin D regulates estrogen synthesis (32). Wang N et al. (33) have shown that vitamin D can also act on the reproductive system. Studies by Dossus L et al. (34) and Van Lenthe FJ et al. (35) on the timing of menarche in girls living in different latitudes found that the higher the latitude, the earlier the age of menarche in girls. It is suggested that the time of sunshine may affect the level of serum vitamin D, and the lack of vitamin D makes the time of menarche earlier in girls. A cohort study of 242 healthy girls aged 5-12 years by Villamor E et al. (14) showed that the time of menarche was earlier in girls with vitamin D deficiency. Villamor believes that obesity is related to vitamin D deficiency and that obese children have early menarche, which suggests that vitamin D may indirectly affect the time of menarche. An alternative view is that vitamin D is negatively correlated with Insulin-Like Growth Factor-I (IGF-1) (32); IGF-1 can affect the onset and progression of precocious puberty by stimulating the release of Progonadoliberin-1 (36); therefore, it is inferred that vitamin D may affect the occurrence of precocious puberty by affecting the production of IGF -1.

There are some limitations that should be considered. First, most of the included studies were performed in China, which limits the generalization of our findings. Having some publications from the Sub-Saharan area of the globe would have made the review an international or global review. Regrettably, we cannot find any related literature from this region which makes it impossible for this review to have a global or international influence. In this part of the world, vitamin D deficiency or insufficiency is endemic. Reviewing studies from this clime may have more impact or may change the final conclusion of the present study. Furthermore, the limited number of participants in some subgroup analyses may lead to unreliable results. Finally, all included studies were case-control studies, which could not determine that vitamin D deficiency was one of the etiology for PP; rather, they concluded there is a correlation between them. A prospective study with a large sample size and much study design is warranted.

This systematic review and meta-analysis demonstrated an association between vitamin D and precocious puberty. However, more high-quality studies, especially prospective cohort studies with big sample sizes or some randomized controlled intervention trials, are needed to validate the reliability of the results. However, considering that vitamin D deficiency is common, in addition to the evidence from this study, adequate vitamin D supplementation is needed to prevent precocious puberty in children. Parents and other guardians are recommended to pay attention to all aspects of early child development and attend regular medical examinations to observe whether there is abnormal development. Child healthcare workers should guide children to supplement vitamin D according to the local situation to effectively improve the needs of the body and promote the healthy growth of children.

## Data availability statement

The original contributions presented in the study are included in the article/supplementary material. Further inquiries can be directed to the corresponding author.

## Author contributions

HC: Conceptualization, Funding acquisition, Writing – original draft. DC: Conceptualization, Writing – original draft. HG: Formal Analysis, Writing – review & editing.
